# The Influence of Serum Uric Acid Level on Non-Motor Symptoms Occurrence and Severity in Patients with Idiopathic Parkinson’s Disease and Atypical Parkinsonisms—A Systematic Review

**DOI:** 10.3390/medicina57090972

**Published:** 2021-09-16

**Authors:** Anna Grażyńska, Klaudia Adamczewska, Sofija Antoniuk, Martyna Bień, Mateusz Toś, Jakub Kufel, Weronika Urbaś, Joanna Siuda

**Affiliations:** 1 Students’ Scientific Association, Department of Neurology, Faculty of Medical Sciences in Katowice, Medical University of Silesia, 40-752 Katowice, Poland; grazynska.anna@gmail.com (A.G.); klaudia.adamczewska75@gmail.com (K.A.); sofija.antoniuk@gmail.com (S.A.); martynabien960@gmail.com (M.B.); 2 Department of Neurology, Faculty of Medical Sciences in Katowice, Medical University of Silesia, 40-752 Katowice, Poland; tosmateusz@gmail.com; 3 Department of Biophysics, Faculty of Medical Sciences in Zabrze, Medical University of Silesia in Zabrze, 41-800 Zabrze, Poland; jakubkufel92@gmail.com; 4 Department of Neurology, St. Barbara Provincial Specialist Hospital No. 5, 41-200 Sosnowiec, Poland; weronika.urbas@op.pl

**Keywords:** Parkinson’s disease, atypical Parkinsonism, uric acid, non-motor symptoms, sleep disorders, cognitive disorders

## Abstract

*Background and Objectives*: A growing number of studies correlated higher levels of serum uric acid (UA) with both: lower risk of Parkinson’s Disease (PD) occurrence and slower progression of the disease. Similar conclusions were made where studies correlated UA with atypical Parkinsonisms (AP) progression. A few researchers have studied the issue of the influence of serum UA on the occurrence of non-motor symptoms (NMS) in PD and AP. Our systematic review is the first review completely dedicated to this matter. *Materials and Methods*: A comprehensive evaluation of the literature was performed to review the relationship between UA and NMS in PD and AP. The systematic review was conducted according to PRISMA Statement guidelines. The following databases were searched starting in April 2021: MEDLINE via PubMed, Embase, and Scopus. During the research, the following filters were used: >2010, articles in English, concerning humans. The study was not registered and received no external funding. *Results*: Seven articles meeting all inclusion criteria were included in this study. Collectively, data on 1104 patients were analyzed. A correlation between serum UA concentration and a few NMS types has been provided by the analyzed studies. In four papers, sleep disorders and fatigue were related to UA for both advanced and early PD. Other commonly appearing NMS domains were Attention/memory (4 studies), Depression/anxiety (3 studies), Cardiovascular (3 studies), Gastrointestinal (1 study), Perceptual (1 study), and Miscellaneous (1 study). For AP, no significant correlation between UA and worsening of NMS has been found. *Conclusions:* Based on the analyzed studies, a correlation between serum UA level and the occurrence and worsening of NMS in PD and APs cannot be definitively determined. Large-scale studies with a more diverse patient population and with more accurate methods of NMS assessment in Parkinsonism are needed.

## 1. Introduction

Parkinson’s Disease (PD) is one of the most common neurodegenerative diseases with unclear and multifactorial pathogenesis. Recent years have brought attention to biological markers which may become future predictors of the disease. One of these markers that are widely discussed in the literature, is the serum uric acid level (UA). UA is considered a strong antioxidant that can protect from PD occurrence. In cell cultures and animal trials, UA prevented the deaths of dopaminergic neurons. Clinical trials suggest that increased UA concentration in serum and cerebrospinal fluid is closely related to slower disease progression, slower motor symptoms progression, and smaller changes in the Unified Parkinson’s Disease Rating Scale (UPDRS) [1,2,3,4,5].

Aside from Parkinson’s disease, several diseases are generally regarded as atypical Parkinsonism (AP). The term ‘atypical Parkinsonism’ is used to describe a class of neurodegenerative disorders that have their own, distinct pathological entities, namely: Multiple System Atrophy (MSA), Progressive Supranuclear Palsy (PSP), Corticobasal Degeneration (CBD), and Dementia with Lewy Bodies (DLB) [6]. Oropesa-Ruiz et al. [7] reported significantly lower serum UA in PSP patients compared to the healthy control. Moreover, Sakuta et al. [8] showed that PD, MSA, and PSP patients have lower serum UA levels. Additionally, for MSA and PSP a significant correlation between UA concentration and disease span and its severity was observed [8]. 

Nonetheless, apart from motor symptoms, non-motor symptoms (NMS) worsen PD and AP patients’ quality of life equally as much, and their specific origin is particularly complex. Dopaminergic pathways dysfunction may affect the occurrence of cognitive disorders, sleep disorders (e.g., RLS), or autonomic disorders (e.g., urinary symptoms) [9]. NMS can be iatrogenic, caused by treatment with dopamine agonists which may, in turn, cause orthostatic hypotension, impulse control disorders, or even hallucinations [10]. Ultimately NMS can be caused by comorbid dysfunctions from several neurotransmitter pathways in the limbic system, such as dopaminergic, noradrenergic, and serotonergic [11]. Contrasting PD, where first NMS are largely unnoticeable and, consequently, omitted, in AP those symptoms often constitute the first signal of developing the disease. Assignment of correct diagnosis may cause a problem when considering that in PD and the diseases that imitate it, both motor and non-motor symptoms are present. The onset of specific NMS, their course, and severity become a widespread suggestion in reconsidering the clinical diagnosis of PD. In recent years it has been suggested that UA concentration may be associated with the occurrence of different NMS and may correlate with higher scores in scales that assess these symptoms: Non-Motor Symptoms Questionnaire (NMS-Quest) and Non-Motor Symptoms Scale of Parkinson’s Disease (NMSS) [12,13]. 

This systematic review aims to organize the existing research concerning the correlation of serum uric acid levels with the occurrence of non-motor symptoms in PD and AP as well as to identify whether uric acid may be a useful marker in predicting and monitoring the occurrence of non-motor symptoms in Parkinsonism.

## 2. Materials and Methods

The systematic review was conducted according to PRISMA Statement guidelines [14]. Supplement S1 constitutes an appendix to this article and is a checklist that proves the reliability of the review. The study was not registered.

### 2.1. Search Strategy and Selection Criteria

**Figure 1:**
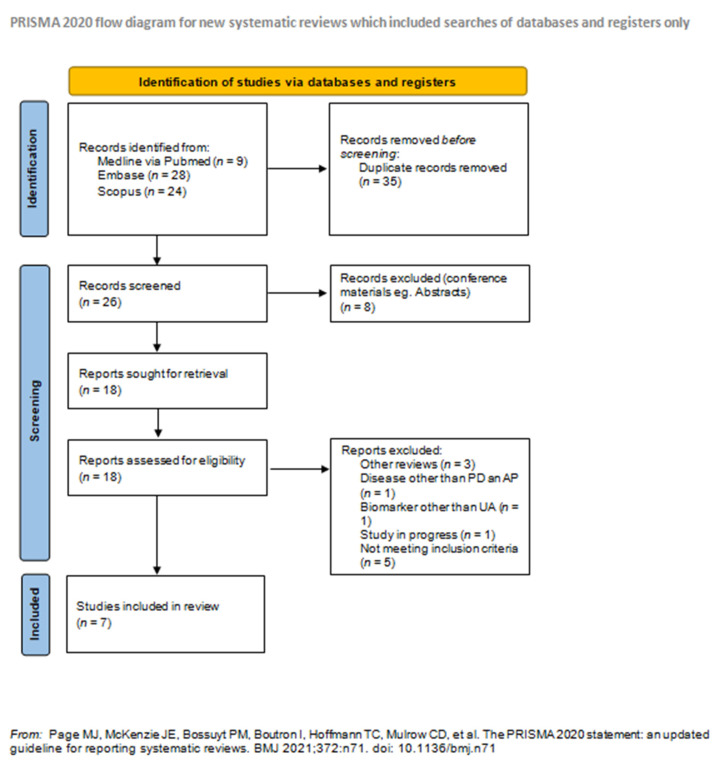
PRISMA flow diagram. For more information, visit: http://www.prisma-statement.org/ (accessed on 15 September 2021).

The following databases were searched in April 2021: MEDLINE via PubMed, Embase, Cochrane, and Scopus. The search was conducted using subsequent MeSH keywords: (Parkinson’s disease) OR (dementia with Lewy bodies) OR (multiple system atrophy) OR (corticobasal degeneration) OR (progressive supranuclear palsy) AND (uric acid)) AND (non-motor symptoms). During the research, the following filters were used: >2010, articles in English, concerning humans. The specific number of articles searched, excluded, and included for review is shown in the PRISMA workflow (Figure 1). 

### 2.2. Data Extraction and Quality Assessment

All of the articles researched in databases (61) were exported and, later, imported to Rayyan QCRI (Qatar Computing Research Institute) where they were subjected to an independent assessment by two reviewers (one neurology resident and one intern, with 5 years of experience in the neurology research club at our center), to avoid the mutual influence on article selection. The articles were appraised according to their usefulness towards this study (articles concerning the topic, containing information about the relation of UA and NMS in PD and/or APs). The criteria for inclusion in the systematic review were: a research study on the relationship of uric acid levels with the occurrence of non-motor symptoms in idiopathic Parkinson’s disease and/or atypical Parkinsonisms (MSA, PSP, DLB, CBD), articles > 2010, articles only in English, studies on humans only. Out of 61 articles, 5 did not meet the inclusion criteria. Duplicate studies (35), other reviews (3), and conference materials, e.g., conference abstracts (8), were removed from our systematic review. In addition, we excluded studies on other neurodegenerative diseases, e.g., Huntington’s disease (1), studies evaluating biomarkers other than UA in which the level of UA was not assessed (1), and studies in progress (1).

All of the conflicts resulting from misunderstanding after unblinding the results of qualifications were settled by a third independent researcher (neurology specialist with 20 years of work experience). In total, 7 articles that met the requirements were qualified for the review. Kappe Coen coefficient was calculated with the result of 0.217 (compliance: 66.66%) indicating fair agreement between the researchers. The studies and methodologies were evaluated, and subgroups based on applied examination protocol, study aim, and outcomes were formed for further analysis. No assumption was made for missing or unclear information and no studies that might appear to meet the inclusion criteria were excluded.

## 3. Results

Seven articles [15,16,17,18,19,20,21] were included in the analysis out of which two [15,19] were prospective studies and five [16,17,18,20,21] were cross-sectional studies (Table S1—Supplementary Material). Huang et al. [15] had a 5-year follow up and Moccia et al. [19] had a 2-year follow-up. The studies were published between 2012 and 2020. In total, 1104 patients took part in the studies (664 male and 450 female) out of which 194 (119 male and 75 female) were included in the prospective studies and 910 (487 male and 423 female) into cross-sectional studies. The mean age of patients from all the publications was 65.32 years. The age range was presented in five studies and varied from 17 to 92 years [16,17,18,19,21]. The average disease span was shown in all studies but two [19,20] and equated to 5.93 years. All of the studies included PD patients and three [15,16,19] described early-stage PD (274 patients). Aside from PD patients, Pan et al. [17] have shown a group of patients with vascular Parkinsonism (VP—80 patients) which were not included in this analysis. Only Chen et al. [20] described MSA patients (47) along with PD patients. No other studies regarding atypical Parkinsonism patients were found. Both of these studies compared their study groups to controls of healthy individuals of similar age. In Chen et al. [20], the control group consisted of 50 patients, whereas Pan et al. [17] did not state the size of the control.

### 3.1. Methodology of Included Studies

#### 3.1.1. Inclusion Criteria

In six of the studies, the main inclusion criterion was a definitive PD diagnosis based on the United Kingdom Parkinson’s Disease Society Brain Bank Diagnostic Criteria (UKPDSBBDC) [16,17,18,19,20,21]. In the study by Huang et al. [15], the inclusion criterion fulfilled the benchmark for PD diagnosis according to the National Institute of Neurological Disorders and Stroke (NINDS). For MSA patients, the inclusion criterion was an unquestionable MSA diagnosis based on the Consensus statement on the diagnosis of multiple system atrophy proposed by Gilman et al. and the criteria proposed by Wenning et al. [20,22,23]. Three studies had additional inclusion criteria: motor symptoms present for no longer than 2 years [15,16,19], no previous treatment with dopaminergic medication [16,19], no severe brain damage in CT and MRI [16,19], communication in English or Mandarin [15]. 

#### 3.1.2. Exclusion Criteria

Three authors excluded all patients with the diagnosis of atypical Parkinsonism, vascular, familial, or secondary Parkinsonism [16,18,19]. Pan et al. [17] and Chen et al. [20] disqualified patients with disabilities caused by neurological disorders other than PD and with any somatic disorder that could influence NMS (e.g., pain syndromes, advanced diabetes mellitus, severe anemia). Three authors [15,17,21] excluded patients with acute and chronic conditions that could be life-threatening (e.g., symptomatic and clinically diagnosed stroke, cancer, failure of any internal organs). Pan et al. [17] and van Wamelen et al. [18] eliminated patients with moderate and severe cognitive disorders according to Mini-Mental State Examination (MMSE; score < 26 points) adjusted to patients’ age and education.

In addition, Huang et al. [15] excluded patients with significant neurological and psychiatric conditions along with patients using medication that could potentially influence UA levels such as diuretics or xanthine oxidase inhibitors.

In both of Moccia et al. studies [16,19], the authors appointed exclusion criteria that could modify serum UA levels: medication (diuretics, NSAID), current smoking, cardiovascular and metabolic disorders, underweight (BMI < 19), and overweight (BMI > 25). Additionally, for one of the studies [19], the authors excluded patients that had ever been treated with anti-Parkinsonian drugs, anticholinergic agents, cholinesterase inhibitors, antidepressants, anxiolytic drugs, or other centrally acting substances that might have affected both motor and non-motor evaluation.

As factors modifying serum UA levels, Chen et al. [20] have determined: hypertension, cerebral ischemia, cardiovascular, diabetes, or renal dysfunction. Furthermore, patients with high prostate carcinoma-related mediators (prostate-specific antigen, carcinoembryonic antigen, or alpha-fetoprotein) were excluded from this study.

Yang et al. [21] have also disqualified all participants with confirmed gastrointestinal tract conditions (e.g., infectious diseases, tumors, non-specific inflammatory bowel disorders) or eating disorders that could potentially influence patients’ nutritional conditions.

#### 3.1.3. Assessment of Disease Advancement

For assessment of disease severity of PD and AP patients the following scales were used: Hoehn Yahr scale (HY), Unified Parkinson’s Disease Rating Scale, Scales for Outcome in Parkinson’s Disease-Motor Scale (SCOPA-Motor Scale), Unified Multiple System Atrophy Rating Scale (UMSARS). The average result on the HY scale was presented in six of the articles and was equal to 2.38 [15,16,17,18,20,21]. UPDRS part III was used in five of the studies [15,16,17,19,21] and the average result obtained by patients was 22.75 points. Van Wamelen et al. [18] used the SCOPA-Motor Scale where the mean result was 14.2 points. UMSARS was used in one study [20] and the average number of points obtained by patients was 36.17 points. Four studies provided mean values of Levodopa Equivalent Daily Dose (LEDD) which was 404.65 mg/day [14,18,19,21].

#### 3.1.4. Means of NMS Assessment

The main tools for NMS assessment were the following questionnaires: Non-Motor Symptoms Scale for Parkinson’s Disease (NMSS) [14,17,18,20] and Non-Motor Symptoms Questionnaire (NMS-Quest) [16,19,21]. In particular, domains of NMSS, a sum of points were taken into account [17,18,20] along with the nine domains which were: Cardiovascular, Sleep/fatigue, Mood, Perceptual, Attention/memory, Gastrointestinal, Urinary, Sexual, Miscellaneous [15,17,18,20]. In Huang et al. [15], NMSS scale domains two and three were not included, since they were assessed using different questionnaires: Montreal Cognitive Assessment (MoCA) and Hospital Anxiety and Depression Scale (HADS). For NMS-Quest, a sum of points was also taken into account [16,19,21] along with domains: Digestive, Urinary, Attention/memory, Hallucinations/delusions, Depression/anxiety, Sexual, Cardiovascular, Sleep, Miscellany [16,19].

The MMSE scale was used by five authors for the assessment of the cognitive function of the patients [16,17,18,20,21]. Additionally, to evaluate cognitive disorders, the authors used the following tools: Wechsler Adult Intelligence Scale 4th Edition (WAIS-IV) digit span backward, Wechsler Memory Scale 4th Edition (WMS-IV) symbol span, frontal assessment battery, fruit fluency, Boston naming test, WAIS-IV similarities, Alzheimer’s Disease Assessment Scale-Cognitive (ADAS-Cog) word list learning with delay recall, Rey–Osterrieth complex figure (ROCF) copying task, ROCF delayed recall, Benton’s judgment of line orientation, 14-items apathy scale, MoCA [15]. To assess mood disorders, authors used HADS [15,18], Hamilton Depression Rating Scale (HAMD), and Hamilton Anxiety Rating Scale (HAMA) [21]. Sleep disorders were evaluated in three studies using the Pittsburgh Sleep Quality Index (PSQI), REM-sleep Behaviour Disorder Single-Question Screen (RBD1Q) [15], and Parkinson’s Disease Sleep Scale-version 1 (PDSS) [18,20]. Other questionnaires used by some of the authors were the 9-items Fatigue Severity Scale [15], UPDRS part I, II, IV [17], Parkinson’s Disease Questionnaire-8 (PDQ-8) [18], and Mini Nutritional Assessment (MNA) [21]. 

#### 3.1.5. Means of UA Measurement

In all studies, UA measurements were made after venous blood samples collection. In the study by Huang et al. [15], serum was separated from blood samples after collection and stored at lower than −80 °C before testing. A professional research laboratory (Quest Laboratories Pte Ltd., Singapore) performed enzymatic testing for the serum UA levels. In two studies by Moccia et al. [16,19], UA was determined by UA2 enzymatic method that used ACN700 reagent kit and COBAS c501 analyzer (Roche Diagnostic, Mannheim, Germany) using serum obtained from fasting blood.

On the other hand, for the Pan et al. study [17], serum was separated by centrifugation at 3000 rpm for 10 min and the isolated sera were stored at −20 °C whilst pending laboratory evaluation. UA levels were measured using the UA test kits with the URO-PAP method (Sinosource Biopharmaceutical Inc., Chengdu, China) with the 7180-ISE Biochemical Analyzer (Hitachi for High-Technology Science Systems Corporation, Tokyo, Japan). In the van Wamelen study [18], the levels of serum UA were obtained from the Hospital database. For Chen et al. [20], venous samples for CRP, Hcy, and UA were obtained from all the patients. A total of 5 mL of blood was drawn from the patients and all measurements were performed three times. Serum was separated by centrifugation at 3000 rpm for 10 min within 1 h. The separated sera were stored at −30 °C until laboratory evaluation. UA levels were measured using UA testing kits with the URO-PAP method (Sinosource Biopharmaceutical Inc.) utilizing a Biochemical Analyzer 7180-ISE (Hitachi High-Technology Science Systems Corporation, Tokyo, Japan). Yang et al. [21] collected samples for routine blood tests (automatic blood cell analyzer, Myry), biochemical analysis, and blood lipid levels (biochemical analyzer, Olympus, Tokyo, Japan).

### 3.2. Results Analysis

Three studies revealed that serum UA levels are related to the number of points in NMS-Quest and NMSS. Moccia et al. [16] have proven that serum UA shows a years’ negative correlation with the sum of NMS-Quest points in both: baseline regression analysis (r^2^ = 0.148; *p* = 0.001) and in an adjusted model taking into account age, sex, disease span, UPDRS part III, HY, and MMSE as accompanying variables (adjusted r^2^ = 0.319; *p* < 0.001). Pan et al. [17] divided serum UA levels into quartiles and observed a significant increase in NMSS points in the lowest quartiles of UA compared to the highest (96.08 vs. 51.28, *p* = 0.003). Furthermore, for patients with more severe NMS burden, lower UA levels were observed; for patients with lower NMS burden, higher serum UA levels (230.26 ± 67.79 mol/L; 314.55 ± 53.76 mol/L, respectively; *p* < 0.001) were observed. Both Pan et al. [17] and van Wamelen et al. [18] showed moderate negative correlation between serum UA levels and NMSS scored in the Spearman’s Rank analysis (rs = –0.310, *p* = 0.005; rs = −0.379, *p* < 0.001, respectively). 

Authors documented a correlation between serum UA and specific domains of non-motor disorders. Huang et al. [15] showed higher UA concentration to be significantly correlated with less severe global cognitive disorders assessed using MoCA (OR = 0.546; *p* = 0.0021), and with lesser fatigue (OR = 0.693; *p* = 0.0408). For Moccia et al. [16], in fundamental logarithmic regression analysis, higher serum UA levels correlated with lesser disability in the following NMS-Quest domains: Attention/Memory (OR = 0.45; *p* = 0.001), Depression/Anxiety (OR = 0.59; *p* = 0.027) and Cardiovascular (OR = 0.29; *p* < 0.001). With application of a model adjusted to age, gender, disease duration, HY, UPDRS part III, and MMSE, a correlation between UA and Attention/Memory (OR = 0.23; *p* = 0.004) and Cardiovascular (OR = 0.11; *p* = 0.009) domains was confirmed and their correlation with age was demonstrated (OR = 1.14; *p* = 0.043, and OR = 0.83; *p* = 0.032, respectively). Additionally, the Sleep domain has proven to be correlated with UA (OR = 0.48; *p* = 0.028). In Pan et al. [17], Sleep/fatigue, Mood, Perceptual, and Gastrointestinal domains have been observed in patients in lower quartiles of serum UA levels compared to those in higher quartiles. Unfortunately, a significant correlation was only shown between serum UA and Sleep/fatigue (rs = −0.393; *p* = 0.001) and Mood (rs = −0.351; *p* = 0.001). On the other hand, van Wamelen et al. [18] described a moderate negative correlation between serum UA and the following NMSS domains: Cardiovascular (rs = −0.285; *p* = 0.008), Sleep/fatigue (rs = −0.299; *p* = 0.005) and Miscellaneous (rs = −0.18; *p* = 0.003). For Moccia et al. [19], the differences between different NMS-Quest domains and serum UA levels were significant in relation to Attention/memory (*p* = 0.0058), Depression/anxiety (*p* = 0.0415) and Cardiovascular (*p* = 0.0007) domains in ANOVA evaluation. Moreover, in post hoc Bonferroni analysis, it was revealed that patients without NMS in Attention/memory, Depression/anxiety, and Cardiovascular domains had significantly higher UA levels than patients with NMS in these domains (*p* = 0.045, *p* = 0.015 and *p* = 0.077, respectively). After adjustment of the results in relation to sex, age, and LEDD, the results were confirmed for Attention/memory (*p* = 0.045) and Cardiovascular (*p* = 0.015) domains.

In his study, Pan et al. [17] divided the population based on sex. The level of serum UA was significantly lower for women than men (246.61 ± 89.04 vs. 314.61 ± 78.41; *p* < 0.001). In the male group, serum UA levels were significantly correlated with the occurrence of Sleep/fatigue (rs = −0.327; *p* = 0.037) and Mood (rs = −0.343; *p* = 0.028) domains. On the other hand, for female group UA was correlated with NMSS scores (rs = −0.341; *p* = 0.034) and Sleep/fatigue (rs = −0.387; *p* = 0.015) and Mood (rs = −0.403; *p* = 0.011) domains.

Moccia et al. [19] defined DNMS as worsening of NMS during patients’ 2-year follow-up. Polynomial regression analysis (categorized into tertials; mean = 0.521; SD = 4.005; IQR 3, +2) between DNMS and UA has shown the lowest tertial to present higher UA levels compared to patients with greater NMS progression, regardless of age, sex and LEDD (OR = 0.488; *p* = 0.023). 

During assessment with different scales determining specific types of NMS, a significant increase in UPRDS part I in lower quartiles of UA serum levels was shown [17]. Huang et al. [15] did not manage to find a correlation between serum UA concentration and any of the 10 detailed scales assessing cognitive abilities of PD patients. Pan et al. [17], on the other hand, has shown a correlation between UA concentration and MMSE points (rs = 0.238; *p* = 0.034). After dividing the patients by sex, this correlation was confirmed only for male population (rs = 405; *p* = 0.009).

Yang et al. [21] has shown that malnutrition is connected to low serum UA (OR = 0.989; *p* = 0.021). Patients with malnutrition risks and malnourishment turned out to have significantly higher results in NMS-Quest (11.56 ± 5.03; *p* < 0.001), MMSE (27.37 ± 2.28; *p* < 0.001), HAMA (11.65 ± 7.89; *p* < 0.001) and HAMD (13.95 ± 9.37; *p* < 0.001).

Chen et al. [20] was the only author to reference patients with atypical Parkinsonism in the form of MSA in his study. Unfortunately, no correlation between serum UA levels and UMSARS (I/II/IV/total), MMSE, a total 2-year NMSS, and any of the nine NMSS domains has been found.

## 4. Discussion

A correlation between serum UA concentration and a few NMS types has been successfully provided by the analyzed studies (Table 1). 

In four papers, sleep disorders and fatigue were related to UA for both advanced and early PD [15,16,17,18]. In their preliminary study, San Martin et al. [24] suggested that increased serum UA can act as a factor modifying neurodegeneration progression in RBD and lowering the rate of transition from RBD to Parkinson’s Disease. Complying with this observation, the authors suggested UA be a useful marker for the assessment of conversion to PD in patients with newly diagnosed RBD. Furthermore, decreased serum UA levels correlated with lower sleep quality, and increased with shorter sleep time in the general population [25,26]. It is important to remember that the sleep domain in both proposed questionnaires—NMSS and NMS-Ques—is broad, consisting of several conditions with differing pathomechanisms such as restless leg syndrome (RLS), REM Sleep Behavior Disorder (RBD), insomnia, or increased daytime sleepiness, and that is why more detailed examinations describing the relation between UA and these disorders (e.g., polysomnography) are needed. Moreover, the analyzed studies found no correlation between UA and specific questionnaires describing sleep disorders in PD such as PDSS, PSQI, or RBD1Q. Fatigue constitutes one of the most important factors influencing the quality of life of PD patients. Accurate pathogenetic mechanisms of the relationship between UA and fatigue in PD are not yet known, however, similar connections were observed in other medical conditions. Decreased serum UA levels were described for patients with chronic fatigue syndrome and acute ischemic stroke, suggesting that low serum UA level relates to decreased ATP synthesis and regeneration [27,28,29].

Another commonly appearing NMS domain was Attention/memory [15,16,17,19] and Perceptual [17]. One of them managed to prove the correlation based on MMSE [17]. In the past, scientists had been showing low serum UA levels to be entailing worse cognitive functions in PD [30]. It is widely acknowledged that cognitive disorders appear in PD along with increasing disease severity. However, studies by Moccia et al. [16,19], that were conducted de novo on a PD population, point not only to the incidence of cognitive disorders for those patients but also to the relationship between cognitive disorders and their progression with serum UA. On the other hand, no correlation between UA and MMSE results was shown, however, patients could report subjective cognitive disorders that were not yet detected by MMSE. Unfortunately, in Huang et al. [15] despite the correlation between UA, cognitive domain, and scores in the MoCA scale, this correlation could not be confirmed in any of the specific tests regarding cognitive abilities. Pan et al. [17] has only verified the relationship between UA and MMSE for a male population which may suggest that UA is not an adequate marker for assessment of memory impairment in women. The results obtained are not a surprise considering that many other studies did not manage to demonstrate the correlation between UA and PD progression for women [31,32,33]. This difference may be due to two reasons: the influence of estrogens on UA metabolism and increased exposure to environmental factors increasing UA for men (bad diet, cigarettes, alcohol).

Studies have also shown a correlation between UA concentration and the Depression/anxiety domain [16,17,19], which suggests UA has a role in both occurrence and progression of mood disorders in PD. Studies are confirming that low levels of serum UA are connected to conditions such as depression, affective bipolar disorder, or social anxiety disorder [34,35,36]. In Moccia et al. [16,19], the relationship between UA and Depression/anxiety domain was not significant for sex, age, and LEDD, and in other studies, a connection between UA and the results of the HADS questionnaire could not be found [15,18]. Meanwhile, Yang et al. [21], where the risk of malnourishment was related to both low serum UA and high NMSS score, has shown higher scores in HAMA and HAMD questionnaires.

A correlation between lowered UA and cardiovascular disorders has also been shown [16,18,19]. High UA levels are related to an increase in cardiovascular risk and in clinical practice their reduction for protection from possible complications such as hypertension is sought after. This disparity can be explained by the fact that the Cardiovascular domain contains symptoms stemming mostly from autonomic system disorders (syncopes, orthostatic hypotension, falls) and classic cardiovascular diseases connected to high UA arise more often from arterial dysfunction and degeneration or destruction of the heart muscle. Furthermore, in the analyzed studies, patients were chosen so that no disturbing factors associated with UA fluctuation such as hypertension, myocardial infarction, or medications could occur. Thus, it can be assumed that high UA levels will act protectively on cardiovascular symptoms in patients with PD.

One study has shown a correlation between UA concentration and the miscellaneous domain where the patients would most often complain of dysosmia and pain [18]. Hyposmia or anosmia occurs in up to 90% of PD patients, however, they were rarely made aware of and that is why could not be caught in the previous examinations. It has been suggested that the presence or progression of hyposmia, along with other early clinical and biochemical markers, could become a marker for an early stage of PD. Furthermore, in the later stages of PD, dysosmia can stem from cholinergic dysfunctions and comorbid dementia. Therefore, for an accurate assessment of the relation between serum UA and dysosmia, more detailed research should be conducted. Interestingly, in this regard, is a report which indicates odor tests with only three smells being as accurate as of the whole odor panel [37,38,39].

Pain accompanies PD patients as often as dysosmia and it does this throughout the whole disease span. The nature of pain, as well as its origin, is complex. Musculoskeletal pains, dystonic pains, central pain syndrome, or peripheral neuropathy are present in patients. Researchers suggest that the cause of pain does not originate in only dopaminergic dysfunction but also dysfunction of other neurotransmitter pathways. Hence, it is essential to seek other biomarkers accompanying the occurrence of pain for its better recognition and more effective treatment [40].

Interestingly, at the beginning of the Moccia et al. [19] study, patients without NMS and with higher levels of serum UA were less prone to NMS development after 2 years of observation, which was not observed in patients with already present NMS. It appears that higher UA levels seem to prevent NMS development provided those symptoms are not yet present. Additionally, it should be taken into account that patients with low UA achieved higher scores in both NMSS and NMS-Quest. For this reason, during the therapeutic process of PD patients, an increase in UA levels should be considered for modification of the course of the disease and decreasing the occurrence of burdensome NMS.

For MSA, no significant correlation between UA and worsening of NMS has been found. In the past, however, studies have shown, that lower levels of serum UA increase the risk of MSA development in male patients [20,41]. Based on this, it could be concluded that UA plays an important role in MSA pathogenesis, but not in the progression of the disease.

The analyzed studies had some limitations, namely: they contained a relatively small patient group, mostly with short disease spans, and only a few included a control group. Secondly, all patients with risk factors commonly occurring in PD and AP modifying UA levels were excluded. There is a concern that by including this patient group the correlations shown would not be as unequivocal. We understand, however, that including patients with additional risk factors would ensue a complicated interpretation and stratification of the data. Furthermore, some studies have excluded patients unable to independently complete the questionnaire which could also influence the results, e.g., in the Attention/memory domain. Additionally, varying methods of UA assessment were used, which is why the results could be incomparable. There are also no studies on other atypical Parkinsonisms except MSA. Above all, the biggest limitation of the analyzed studies was the use of questionnaires that only allow subjective diagnosis and screening of NMS, such as NMSS and NMS-Quest. Some authors have also used questionnaires dedicated to specific NMS such as HAMA and HAMD, however, there is a chance that those were insufficient for particular non-motor symptoms assessment. In this regard, an interesting project to consider is Cohort of Patients with Parkinson’s Disease in Spain, 2015 study (COPPADIS-2015) which is due to finish in 2022, and which could be a prospective, multicenter, non-invasive, long-term study of the progression of PD. The project is based on four aspects: PD as a global disease (assessment of motor and non-motor symptoms), quality of life and caretakers’ problems, biomarkers, progression of the disease. This type of holistic view on PD, including the relation of biomarkers and NMS, seems very promising [42]. 

Our systematic review had some limitations. First of all, a small number of studies, with a relatively small number of patients, were qualified for the study. Only one out of seven studies described the relationship of UA level with NMS in atypical Parkinsonism. In addition, this study only reported MSA with the omission of other forms of AP. Due to a small number of studies, we were not able to perform a meta-analysis. More data coming from future research is needed.

## 5. Conclusions

Correspondingly high serum uric acid levels have been identified as a molecular predictor of both reduced risk of disease development, slow progression of idiopathic Parkinson’s disease, and atypical Parkinsonism. Numerous studies have confirmed the role of UA as an antioxidant and indicate its neuroprotective effect in the pathogenesis of neurodegeneration in PD and AP. In the case of non-motor symptoms in PD, the studies we analyzed showed a correlation between some NMS domains and serum UA concentration. The most numerous correlations were confirmed for the domain of sleep/fatigue and attention/memory. Other domains that were found are cardiovascular, mood, perceptual, gastrointestinal, and miscellany. As also shown by the studies of Moccia et al. [16,19], early Parkinsonian patients who had high levels of serum UA developed NMS less frequently. In clinical practice, it can be assumed that patients with low UA will need more frequent monitoring of NMS incidence, with commonly available scales or specialized tools. However, it should be remembered that the level of UA does not make it possible to distinguish PD and AP from one another, and it also does not allow to predict which non-motor symptom will occur in a given patient. In turn, UA itself remains a biomarker susceptible to the influence of many factors, such as drugs and comorbidities. Too high AU level is also a risk factor for cardiovascular disease. In the future, more research is needed on an appropriate UA concentration threshold that will be both neurologically and cardiologically safe. Moreover, more specific and detailed methods of particular NMS assessment should be used in future studies’ design, as well as engaging specialists from other fields of medicine such as psychiatry, cardiology, or geriatrics. Furthermore, larger, more diverse patient populations should be used and more detailed studies on the correlation between UA and NMS in other Parkisnonisms.

## Figures and Tables

**Table 1:** Individual domains and connections with uric acid.

Domain in NMSS (NMS-Quest)	Huang X. et al. [15]	Moccia M. et al. [16]	Pan M. et al. [17]	van Wamelen et al. [18]	Moccia M. et. al. [19]	Chen D. et al. [20]	Yang T. et al. [21]
Total NMSS (NMS-Quest)	-	+	+	+	-	-	-
Cardiovascular (Cardiovascular)	-	+	-	+	+	-	-
Sleep/fatigue (Sleep)	+	+	+	+	-	-	-
Mood (Depression/anxiety)	-	+	+	-	+	-	-
Perceptual (Hallucinations/delusions)	-	-	+	-	-	-	-
Attention/memory (Attention/memory)	+	+	+	-	+	-	-
Gastrointestinal (Gastrointestinal)	-	-	+	-	-	-	-
Urinary (Urinary)	-	-	-	-	-	-	-
Sexual (Sexual)	-	-	-	-	-	-	-
Miscellany (Miscellany)	-	-	-	+	-	-	-

Abbreviations: NMSS, Non-motor Symptoms Scale; NMS-Quest, Non-motor Symptoms Questionnaire. “+”—correlation or statistical significance; “-”—no correlations or statistical significance.

## Data Availability

Search results are available from the authors.

## Supplementary Materials

The following are available online at https://www.mdpi.com/article/10.3390/medicina57090972/s1, Supplement S1: PRISMA 2020 Checklist, Supplement S2: Queries to the searched databases with filters, Table S1: Detailed information about the demography of participants and aims of studies.
