# Supplementary material for: The Influence of Serum Uric Acid Level on Non-Motor Symptoms Occurrence and Severity in Patients with Idiopathic Parkinson’s Disease and Atypical Parkinsonisms—A Systematic Review
Source: Medicina (Kaunas). 2021 Sep 16;57(9):972. doi: 10.3390/medicina57090972 (PMC8468188; doi:10.3390/medicina57090972)
Supplement: Supplementary file 1 [file medicina-57-00972-s001.zip › medicina-1358356-supplementary/Supplementary Materials/Supplement S2.pdf]

Supplement S2. Queries to the searched databases with filters.

PubMed

Filters: abstract, only human, <10 years

("parkinson disease"[MeSH Terms] OR ("parkinson"[All Fields] AND "disease"[All Fields]) OR "parkinson disease"[All Fields] OR "parkinson s disease"[All Fields] OR ("lewy body disease"[MeSH Terms] OR ("lewy"[All Fields] AND "body"[All Fields] AND "disease"[All Fields]) OR "lewy body disease"[All Fields] OR ("dementia"[All Fields] AND "lewy"[All Fields] AND "bodies"[All Fields]) OR "dementia with lewy bodies"[All Fields]) OR ("multiple system atrophy"[MeSH Terms] OR ("multiple"[All Fields] AND "system"[All Fields] AND "atrophy"[All Fields]) OR "multiple system atrophy"[All Fields]) OR ("corticobasal"[All Fields] AND ("degenerate"[All Fields] OR "degenerated"[All Fields] OR "degenerately"[All Fields] OR "degenerates"[All Fields] OR "degenerating"[All Fields] OR "degeneration"[All Fields] OR "degenerations"[All Fields])) OR ("supranuclear palsy, progressive"[MeSH Terms] OR ("supranuclear"[All Fields] AND "palsy"[All Fields] AND "progressive"[All Fields]) OR "progressive supranuclear palsy"[All Fields] OR ("progressive"[All Fields] AND "supranuclear"[All Fields] AND "palsy"[All Fields]))) AND ("uric acid"[MeSH Terms] OR ("uric"[All Fields] AND "acid"[All Fields]) OR "uric acid"[All Fields]) AND ("non-motor"[All Fields] AND ("diagnosis"[MeSH Subheading] OR "diagnosis"[All Fields] OR "symptoms"[All Fields] OR "diagnosis"[MeSH Terms] OR "symptom"[All Fields] OR "symptom s"[All Fields] OR "symptomes"[All Fields]))

Embase

Filters: abstract, only human, <10 years

('parkinsons disease'/exp OR 'parkinsons disease' OR (parkinsons AND ('disease'/exp OR disease)) OR 'dementia with lewy bodies'/exp OR 'dementia with lewy bodies' OR (('dementia'/exp OR dementia) AND with AND lewy AND bodies) OR 'multiple system atrophy'/exp OR 'multiple system atrophy' OR (multiple AND system AND ('atrophy'/exp OR atrophy)) OR 'corticobasal degeneration'/exp OR 'corticobasal degeneration' OR (corticobasal AND ('degeneration'/exp OR degeneration)) OR 'progressive supranuclear palsy'/exp OR 'progressive supranuclear palsy' OR (progressive AND supranuclear AND ('palsy'/exp OR palsy))) AND ('uric acid'/exp OR 'uric acid' OR (uric AND ('acid'/exp OR acid))) AND ('non-motor symptoms' OR ('non motor' AND ('symptoms'/exp OR symptoms)))

Scopus

Filters: abstract, only human, <10 yers, articles, review

parkinson's AND disease OR dementia AND with AND lewy AND bodies OR multiple AND system AND atrophy OR corticobasal AND degeneration OR progressive AND supranuclear AND palsy AND uric AND acid AND non-motor AND symptoms AND (LIMIT-

TO (PUBSTAGE , "final")) AND (LIMIT-
TO (PUBYEAR , 2020) OR LIMIT-
TO (PUBYEAR , 2019) OR LIMIT-
TO (PUBYEAR , 2018) OR LIMIT-
TO (PUBYEAR , 2017) OR LIMIT-
TO (PUBYEAR , 2016) OR LIMIT-
TO (PUBYEAR , 2015) OR LIMIT-
TO (PUBYEAR , 2014) OR LIMIT-
TO (PUBYEAR , 2012) OR LIMIT-
TO (PUBYEAR , 2011)) AND (LIMIT-
TO (EXACTKEYWORD , "Human")) AND (LIMIT-
TO (LANGUAGE , "English"))
