# Supplementary material for: The Influence of Serum Uric Acid Level on Non-Motor Symptoms Occurrence and Severity in Patients with Idiopathic Parkinson’s Disease and Atypical Parkinsonisms—A Systematic Review
Source: Medicina (Kaunas). 2021 Sep 16;57(9):972. doi: 10.3390/medicina57090972 (PMC8468188; doi:10.3390/medicina57090972)
Supplement: Supplementary file 1 [file medicina-57-00972-s001.zip › medicina-1358356-supplementary/Supplementary Materials/Table S1.pdf]

Table S1. Detailed information about the demography of participants and aims of studies.

Study	Year	Type of study	Number of participants	Number of observers	Sex (Male; Female)	Described diseases	Stage of disease	Age [years]	Age range [years]
Huang X.et al. [15]	2018	Prospective study (5 years follow-up)	125	2	(75; 50)	PD	early stage only	63.47 ± 8.99	no data
Moccia M. et al. [16]	2014	Cross-sectional study	80	a few physicians	(51; 29)	PD	early stage only	59.3 ± 7.9	42-74
Pan M. et al. [17]	2012	Cross-sectional study	80	a few physicians	(41; 39)	PD, VP (excluded)	no data	60.25±12.24	17-88
van Wamelen. et al. [18]	2020	Cross-sectional study	87	no data	(64; 33)	PD	no data	68.34±10.34	no data
Moccia M. et. al. [19]	2015	Prospective study (2 years follow-up)	69	a few physicians	(44; 25)	PD	early stage only	59.5 ± 8.3	44-74
Chen D. et al. [20]	2015	Cross-sectional study	107 (60 PD, 47 MSA)	a few physicians	PD: (34; 26), MSA: (31; 16)	PD, MSA	no data	PD: 63.10 ± 10.62, MSA: 58.74 ± 10.18	PD: 41-80, MSA: 23-79
Yang T. et al. [21]	2020	Cross-sectional study	556	no data	(324; 232)	PD	all stages	68.37 ± 10.47	36-92

Study	Disease duration [years]	Hoehn-Yahr scale (mean points)	UPDRS part III (mean points)	Other scales (mean points)	LEDD [mg]	Aim of the study
Huang X.et al. [15]	4.19 ± 3.44	1.91 ± 0.36	21.30 ± 10.19	no data	210.64 ± 152.38	Investigate the relation between serum UA and motor subtypes of PD and NMS in the early PD stage.
Moccia M. et al. [16]	1.1 ± 0.48	2	15.25 ± 7.7	no data	no data	Uncovering the correlation between serum UA and NMS occurrence in de novo PD. Settling if serum UA could be a useful marker for early NMS occurrence
Pan M. et al. [17]	4.10 ± 3.65	2.21 ±1.12	22.90 ± 9.77	no data	no data	Comparison of serum UA in healthy individuals with PD and VP comparing age, disease duration, motor dysfunctions and NMS between PD and VP patients assessing whether UA levels in PD and VP are related to low motor function and levodopa dosage. Inspect the presence of correlation between serum UA and incidence of specific domains in NMSS.
van Wamelen. et al. [18]	11.92 ± 6.50	3	no data	SCOPA-Motor Scale: 14.20±6.74	634.97 ± 542.25	Investigate the correlation between serum UA levels and higher frequency of NMS incidence in a larger and more diverse cohort of PD patients (using structured outcomes for NMS and motor scores).
Moccia M. et. al. [19]	no data	no data	15.6 ± 7.7	no data	339.5 ± 136.9	Assessing the usefulness of baseline UA levels as a marker for progression of newly diagnosed PD evaluating the relation between baseline UA levels and progression of motor dysfunctions and requirement of antiparkinsonian drugs.
Chen D. et al. [20]	no data	PD: 2.18 ± 0.95, MSA: 3.21 ± 0.81	no data	UMSARS: 36.17 ± 10.49	no data	Examining whether inflammatory mediators such as CRP, homocysteine (Hcy) and UA influence the severity and frequency of MSA in comparison to PD and control. Assessing if serum CRP,

Yang T. et al. [21]	6.34 ± 4.80	2.41 ± 0.73	25.02 ± 12.37	no data	420.32 ± 231.52	Hcy and UA in MSA patients are associated with motor and non-motor symptoms and specific NMSS domains. Determining the diagnostic values of serum CRP, Hcy and UA for MSA. Evaluation of connection between patients' nutritional status and progression of PD.

Abbreviations: PD, Parkinson's Disease; MSA, multiple system atrophy; VP, vascular parkinsonism; UPDRS, Unified Parkinson's Disease Rating Scale; LEDD, Levodopa Equivalent Daily Dose; UA, uric acid; NMS, non-motor symptoms; CRP, C-reactive protein; Hcy, homocysteine.
